# Grain Amaranths Are Defoliation Tolerant Crop Species Capable of Utilizing Stem and Root Carbohydrate Reserves to Sustain Vegetative and Reproductive Growth after Leaf Loss

**DOI:** 10.1371/journal.pone.0067879

**Published:** 2013-07-04

**Authors:** Erandi Vargas-Ortiz, Eduardo Espitia-Rangel, Axel Tiessen, John Paul Délano-Frier

**Affiliations:** 1 Unidad de Biotecnología e Ingeniería Genética de Plantas. Centro de Investigación y de Estudios Avanzados-Unidad Irapuato, Irapuato, Guanajuato, México; 2 Instituto Nacional de Investigaciones Forestales, Agrícolas y Pecuarias, Celaya, Guanajuato, México; Key Laboratory of Horticultural Plant Biology (MOE), China

## Abstract

Tolerance to defoliation can be defined as the degree to which productivity is affected by photosynthetic area reduction. This trait was studied in grain amaranth (*Amaranthus cruentus* and *A. hypochondriacus*), which are considered to be a highly defoliation-tolerant species. The physiological and biochemical responses to increasing levels of mechanical leaf removal up to total defoliation were quantified. Tolerance appeared to be dependent on various factors: ( i) amount of lost tissue; (ii) mechanics of leaf tissue removal; (iii) environment, and (iv) species tested. Thus, grain amaranth was found to be a highly tolerant species under green-house conditions when leaf tissue loss was performed by gradual perforation. However, tolerance was compromised under similar conditions when defoliation was done by gradual cutting of the leaf. Also tolerance in completely defoliated plants tended to decrease under field conditions, where differences between *A. cruentus* and *A. hypochondriacus* were observed. All non-structural carbohydrate (NSC) levels were reduced in stems and roots of totally defoliated amaranths one day after treatment. Such depletion probably provided the carbon (C) resources needed to sustain the early recovery process in the absence of photosynthetic capacity. This was corroborated by shading of intact plants, which produced the same rapid and drastic reduction of NSC levels in these tissues. These results emphasize the role of stored NSCs, particularly starch, in buffering the impact of severe defoliation in amaranth. The fall in sucrose synthase and cell wall invertase activity observed in stems and roots soon after defoliation was consistent with their predicted shift from sink to source tissues. It is concluded that mobilization of C stores in stems and roots, is a physiologically important trait underlying tolerance to defoliation in grain amaranth.

## Introduction

Tolerance has been defined as the degree to which a plant can maintain the same level of reproductive success under an adverse environment compared to non-limiting conditions [1], [2]. Tolerance reflects the capacity of a crop to allocate carbon (C) to different organs and to produce seeds despite limited photosynthesis caused by various circumstances, including pathogen attack [3], viral infection [4], water stress [5], salt stress [6], [7], soil nutrient limitations [8], shading [9] or the loss of leaf area [10], [11]. Defoliation tolerance (DT) is therefore relevant for (a)biotic stress research [12], [13], [14].

Several mechanisms have been associated with increased tolerance, including elevated rates of photosynthesis in remaining leaves of partially defoliated plants, re-growth stimulation and increased branching through the release of apical dominance, alteration of phenology or plant architecture, utilization of high pre-damage stored C resources or the ability to reallocate them to less vulnerable tissues, re-sorption of nutrients from senescent/damaged leaves and higher reproductive efficiency through increased percentage of fruit/seed set [15]. Such physiological strategies reflect altered resource partitioning among sink and source tissues, which is inherently related to osmotic adjustment, phloem physiology and carbohydrate metabolism (i.e. reversible inter-conversion between sucrose, hexoses and starch) [16], [17], [18], [19]. Recent investigations have also begun to reveal some biochemical and genetic mechanisms triggered in response to defoliation in diverse plant species [20], [21], [22], [23]. In *Sesbania* and *Populus*, physical leaf damage alters the pattern of resource allocation to various vegetative and reproductive organs [24], [25]. However, in annual crop plants there is still rather limited biochemical information regarding how these metabolic changes occur simultaneously in various organs such as roots, stems, sink leaves, flowers and seeds after the removal of source leaves.

Grain amaranths (predominantly *Amaranthus cruentus, A*. *hypochondriacus* and *A. caudatus*) are dicotyledonous plants that have not yet been subjected to intense breeding [26]. They are C4 photosynthesis type plants widely distributed in the subtropical and temperate areas of the world [26], [27] with the ability to grow in poor soils under unfavorable environmental conditions, surviving low water availability, high light intensity and extreme temperatures [28], [29]. A number of species are ubiquitous weeds (e.g. *A. spinosus* and *A. retroflexus*), whereas others (*A. cruentus, A. dubius, A hybridus, A. lividus, A. mantegazzianus* and *A. tricolor*), are used as foliar vegetables, because the leaves are readily edible due to their high vitamin and mineral content [30], [31], [32]. Compared to cereals, amaranth seeds are notable for their high contents of gluten-free protein having a nutritionally balanced amino-acid composition [26], [33], [34]. Moreover, there is a growing awareness of the health-promoting properties of amaranth grain proteins and oil, which may be used for the prevention of some types of cancer, hypertension and high-lipid related disorders [35].

In this work, the morphological, physiological and biochemical responses of grain amaranth to increasing degrees of mechanical leaf removal are described. It is shown that grain yield in amaranth was not reduced after the mechanical removal (by hole punching) of 20-to-100% of its leaves at the pre-flowering stage. This study complemented previous reports describing the high tolerance to defoliation shown by grain amaranths [20], [36]. Moreover, the results herewith shown indicate that tolerance may be genetically determined, since different degrees of tolerance between two grain amaranth species examined were observed. It is also shown that in highly defoliated amaranths, extensive utilization of carbohydrate reserves, involving mostly starch and sucrose, occurred in stems and roots during the early recovery process in which photosynthetic activity was absent or greatly limited due to the loss of 50-to-100% of foliar tissue. This was accompanied by an arrest of root growth and by a sharp reduction in stem and root sucrose synthase (SUS) and cell wall invertase (CWI) activities, which probably reflected their shift from sink to source tissues. Increased amylase (AMY) activity was also observed at this stage. It is proposed that this process provides the resources to support regrowth, inflorescence development and viable seed production. This was corroborated by shading experiments, which caused a similarly drastic and rapid decline in non-structural carbohydrates (NSC) reserves, including starch, in stem and roots and also in leaves of intact plants. The study hereby provides data that allows a better understanding of the biochemical mechanism(s) underlying defoliation stress tolerance in highly defoliation-tolerant species, such as grain amaranth.

## Materials and Methods

### Plant materials and growth conditions

The Mexican varieties “*Tarasca*”, “*Dorada*” and “*Amaranteca*” classified as *A*. *cruentus*, and “*Nutrisol*” “*Gabriela*” and “*Revancha*”, classified as *A. hypochondriacus*, were used in the greenhouse and/or field experiments here described. All experiments were performed in April to October, which is the optimal growth season for grain amaranth cultivation in Mexico. For the greenhouse experiments, *A. cruentus* var. *Tarasca* seeds were germinated in 60-space germinating trays as described previously [28]. The trays were maintained in a growth chamber kept at 26°C and 75% relative humidity (R.H.). Amaranth plantlets were subsequently transplanted to 12-L plastic pots, containing a sterile substrate [20], 21-days after germination. They were fertilized once, one week after transplant, with 400 mL of a 20: 10: 20 (N: P: K) nutrient soil drench solution prepared according to the manufacturer's instructions (Peters Professional; Scotts-Sierra Horticultural Products, Marysville, OH, USA). The plants were subsequently transferred to a commercial green house with zenithal and lateral type ventilation (Baticenital 850; ACEA S.A., Mexico) in which all experiments were performed within a 10°C (night) to 35°C (day) temperature range, an average 55% R.H. and under natural light (∼1300 μE, ∼12 h photoperiod). For the field experiment, seeds were germinated in 100-space germinating trays filled with a sterile substrate under greenhouse conditions. A chemical analysis of this substrate showed that it was highly fertile, with higher than average levels of available nitrogen, phosphorus and potassium (data not shown).

### Field experiments

The experiments were performed at the experimental field of The National Institute for Research in Forestry, Agriculture and Livestock (INIFAP), situated in Celaya, Gto., México (20° 31′ 44″ N, 100° 48′ 54″ W). Four Mexican varieties were used in the experiment: “*Dorada*” and “*Amaranteca*” and “*Nutrisol*” and “*Revancha*”. Seeds were germinated in June, 2011. Seedlings were directly transplanted to the damp field on July 29 2011. Fertilization with 60-40-30 kg/ha (N-P-K) was applied at sowing; 130 kg/ha of urea were subsequently applied 40 days later to provide a total of 120 kg N per ha. Each individual variety was established in four plots of four rows per plot. Each row was 5 m long, and the separation between plants was 10 cm. The defoliation treatments (50 or 100% defoliation) were only performed with plants situated in the central rows, and then with only half of the population in each row. Plots were randomly distributed in the field. Defoliation treatments were done at the pre-flowering stage, which varied with each different variety, but occurred within 19 (for “*Revancha*”), 26 (for “*Dorada*”), 29 (for “*Amaranteca*”) and 33 days (for “*Nutrisol*”) after transplantation.

Defoliation was performed by cutting with scissors, from the apex, one third of the leaf tissue required to achieve the desired defoliation levels (50 and 100% defoliation, respectively) during 3 successive days. Cutting of leaf tissue was always done in the morning. In fully defoliated plants, the residual 1/3 of leaf tissue that still remained attached to the plant after two days was simply removed from the plant on the third day by cutting it from the base of the petiole.

Harvest was started on November 1^st^, 2011. Five plants per treatment in each plot were sampled. These were randomly placed in the central sections of the rows. Selected plants were individually measured for height and were subsequently cut at the base of the stem for drying under natural greenhouse conditions to obtain the dry weights of shoots and panicles. Yields were measured after carefully threshing the grain from the dry panicles.

### Green house Experiments

Three defoliation experiments were performed in the years 2010 (1) and 2011 (2) in the above greenhouse localized at Cinvestav-Irapuato, México, (20° 40′ 18″ N, 101° 20′ 48″ W) with 45-day-old *A. cruentus* “*Tarasca*” plants. At this point all plants were at the pre-flowering stage. Four groups of 30 plants were formed. These were used to apply four different defoliation treatments: intact, 20%, 50% and 100% defoliation. The desired defoliation levels, done by perforation of the leaves with a hole-puncher, were gradually obtained, as above, within a three-day period. Thus, by the third day the 20% and 50% defoliation treatments were complete, whereas in the fully defoliated plants, the remaining leaf blades that had previously lost ∼80% of their surface were separated from the plants by cutting them from the plant at the base of the petiole. Ten plants per group were sampled at 1, 30 and 110 days after defoliation (dad). Phenological parameters such as plant height, root, and shoot fresh weight, and stem thickness were taken at each time point. Samples of root and stem tissues were also taken, they were flash frozen in liquid nitrogen and later freeze dried for NSC measurements and enzymatic determinations. Fresh panicle weights were measured at 110 dad, and also after drying. Seed yield was determined from seeds recovered from dry panicles. Seed samples were also subjected to different analyses (see below). A fourth greenhouse experiment was performed in parallel with the field experiment described above, with two modifications: i) six cultivars were examined instead of the four used in the field and, ii) only 100% defoliation treatments, done by cutting with scissors, starting from the apex of the leaves, were applied.

### Shading experiments

Leaf shading treatments were performed in the green house and were used to perturb the source-sink balance in 45-days-old “*Tarasca*” (*A. cruentus*) plants by covering the plants with progressively denser sunlight-blocking black knitted shade cloth-screens (Agroriego, Mexico) for three days. Plants were covered with a 20% screen at day 1, followed by a 50% screen at day 2 and with an 80% screen at day 3. Control plants remained un-shaded for the duration of the experiment. Light conditions were checked daily throughout the experiment using a LI-6400 portable photosystem unit (LI-COR Biosciences Inc., Lincoln, NE, USA) to measure the gradual reduction in levels of light exposure. A parallel defoliation experiment was performed in which plants were 100% defoliated by leaf apex cutting as described above.

### Measurement of non-structural carbohydrates

Soluble sugars were extracted from each tissue according to adapted methodologies [37], [38]. Briefly, 50 mg of ground vacuum dried tissue was extracted in 50 mM Hepes KOH (pH 7.4); 5 mM MgCl_2_, 80% ethanol, three times at 80°C. Soluble extracts were combined and assayed enzymatically for sucrose (SUC), glucose (GLC) and fructose (FRC) in a microplate format [39]. The insoluble starch pellet was dissolved in 0.5 ml 10 mM KOH at 99°C and autoclaved for 30 min. Starch was hydrolyzed in 50 mM Hepes (pH 7.5) at 37°C overnight by the addition of 10 units of α-amylase (EC 3.2.1.1; Roche) and 10 units of amyloglucosidase (EC 3.2.1.3; Roche). Samples were centrifuged (13000×g for 5 min), the resulting supernatant was stored at 4°C and the pellet was hydrolyzed again for 30 min at 37°C. Both supernatants were combined and an aliquot was enzymatically assayed for GLC, as above.

### Quality of amaranth seeds harvested from defoliated plants

In order to detect differences between seeds from intact and defoliated plants, the following parameters were evaluated: i) color and shape of seeds; ii) average weight of seeds; iii) germination efficiency, and iv) total protein [40], starch [37] and lipid composition. Seeds were manually separated from the dry panicle, as above. One hundred seeds were obtained from ten panicles per treatment, in triplicate, and the weight was averaged. Total lipids were determined gravimetrically as follows: 500 mg of flour was extracted three times with 1 ml hexane at 99°C for 10 min. Samples were centrifuged at 13000×g for 5 min and recovered in a pre-weighted glass vial. Hexane was vacuum evaporated and remaining lipids were quantified with an analytical balance. Total protein was extracted using the de-fatted flour [41].

### Invertase, sucrose synthase and amylase activities in vitro

Acid soluble (vacuolar) and insoluble (cell wall), neutral (cytoplasmic) invertase and sucrose synthase activities were determined as described in [38] at an optimum pH of 5.5, 5.0, 7.0 and 8.0 respectively. Total amylolytic activities were determined as described previously [42], [43].

### Statistical procedures

All experiments were conducted using a randomized complete block design with an adequate number of replicas. If not otherwise indicated, ten plants per sampling time were employed. One-way ANOVAs were utilized to evaluate differences between treatment means. For ANOVAs where the F test was significant at *P* = 0.05 or lower, the Dunnett test was applied. Statistical analysis was performed with R version 2.15.3 [44].

## Results

### Amaranth's response to defoliation in the greenhouse

**Figure 1:**
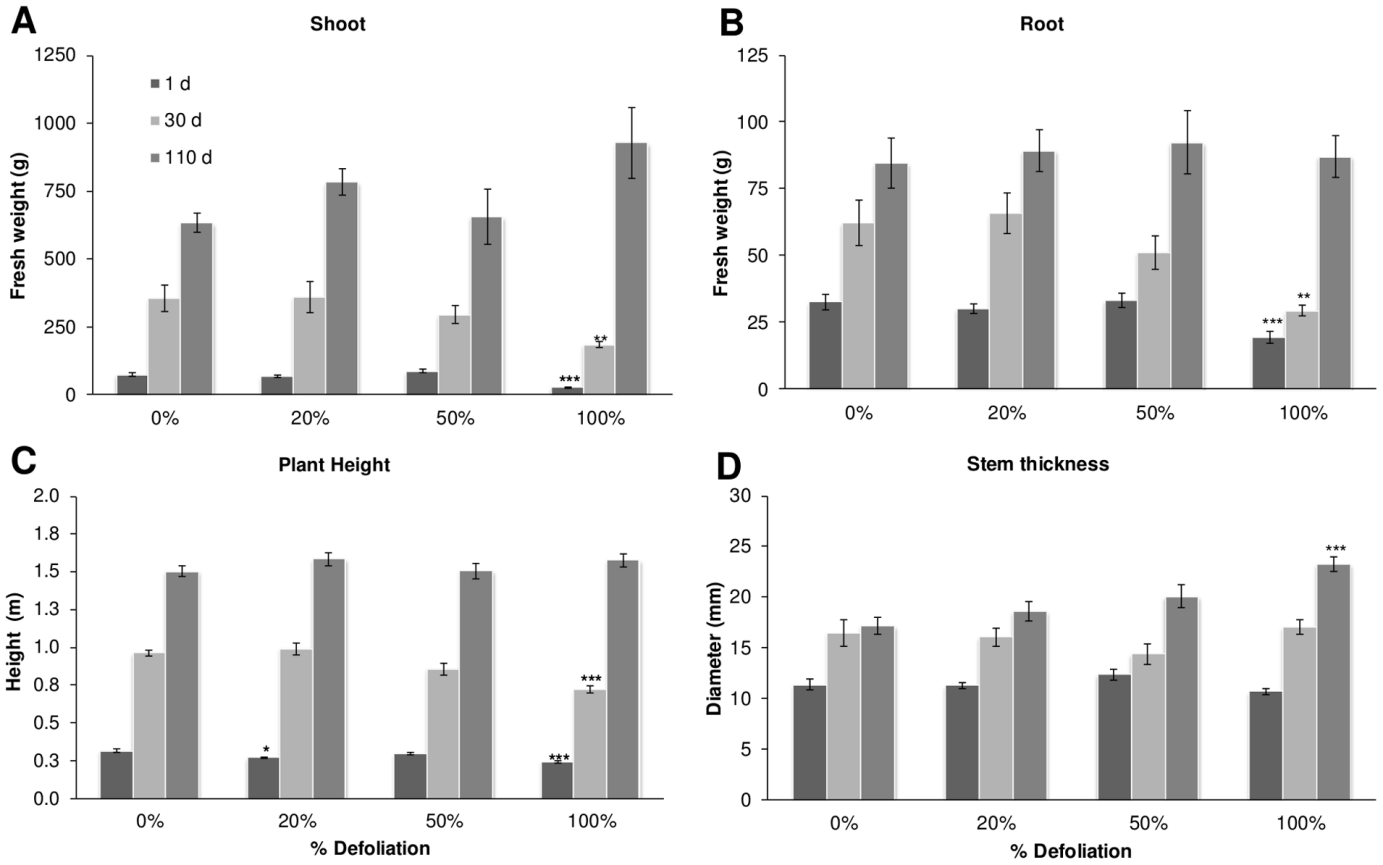
Effect of defoliation on phenological parameters in grain amaranth grown in the greenhouse. 45-day-old *A. cruentus* plants were subjected to 4 defoliation degrees: 0%, 20%, 50% and 100% Phenological parameters, A shoot; B root, C plant height, and D stem thickness, were measured at 1, 30 an110 d after treatment. Each bar represents the mean ± SE (n = 10). The asterisks over the bars represent statistical significance at * *p* = 0.05, ** *p* = 0.01, *** *p* = 0.001 for the Dunnett test.

Three controlled defoliation experiments were performed in the greenhouse with *A. cruentus* cv. *Tarasca*, a Mexican variety which is well adapted to the “Bajío” region of central México that includes Celaya and Irapuato, where the experiments were localized [45]. Forty five-day-old plants were grown in 12-L plastic pots and were subjected to three defoliation intensities: 20%, 50% and 100% of foliar tissue loss by perforation. Plants were then harvested at different times after defoliation (1, 30 and 110 dad). Interestingly, most growth parameters evaluated (shoot and root biomass and plant height) were negatively affected only in the completely defoliated plants, and only at 1 and 30 dad (Figure 1 A, B and C). A temporary arrested growth observed between 1 and 30 dad in roots and shoots of 50% and 100% defoliated plant could have been an indication that C reserves were being exported to other sinks, such as leaf meristems, and emerging young leaves, rather than being used for their own growth.

**Figure 2:**
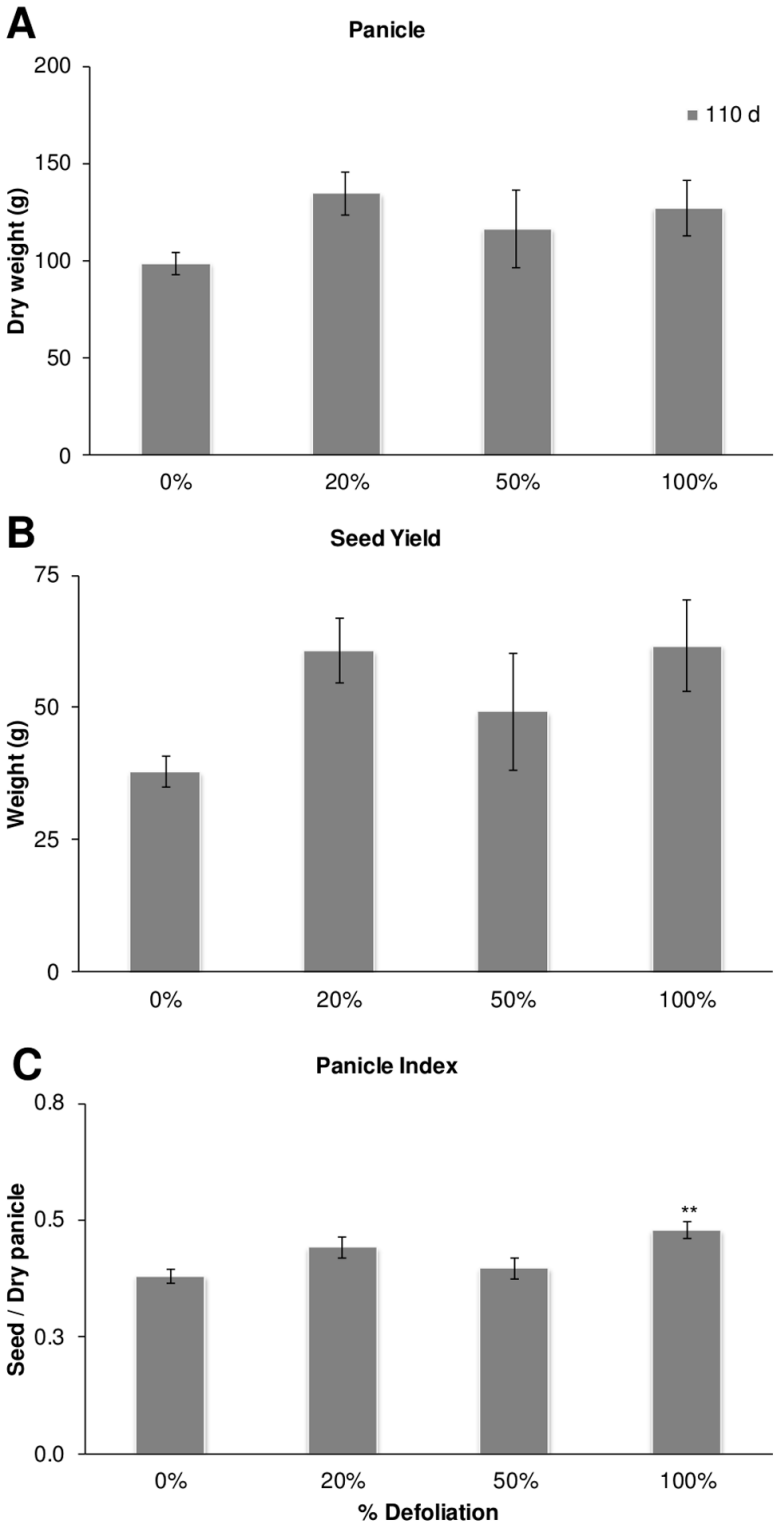
Effect of defoliation on amaranth's reproductive yield. A Panicle dry weight. B Seed yield at maturity, C Calculated panicle index as the ratio of seed weight and panicle weight. Each bar represents the mean ± SE (n = 10). The asterisks over the bars represent statistical significance at * *p* = 0.05, ** *p* = 0.01, *** *p* = 0.001 for the Dunnett test.

**Figure 3:**
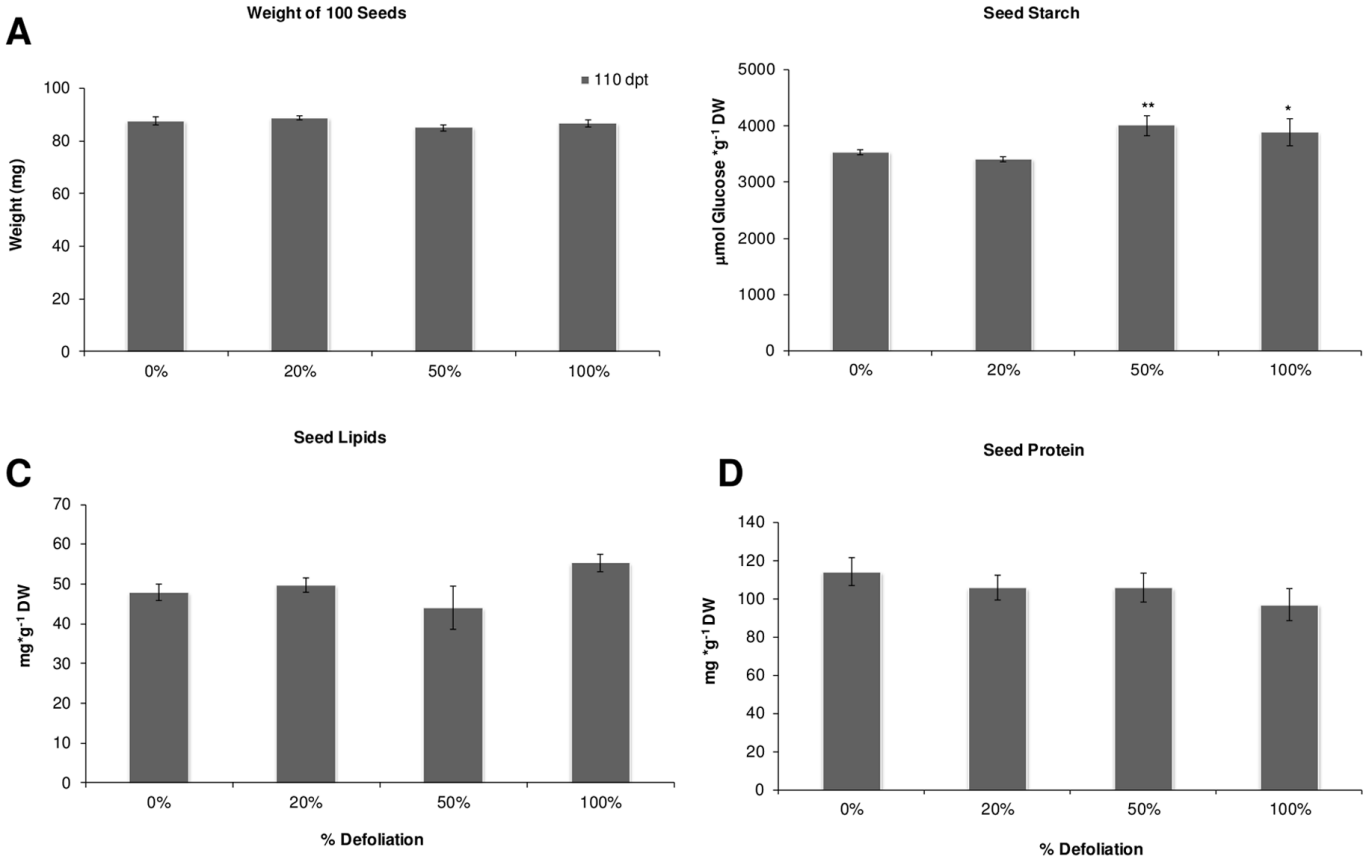
Seed composition in defoliated amaranth. 110 d after defoliation amaranth seeds were collected and analyzed for A 100 seeds weight, B seed starch, C seed lipids and D seed protein. Each bar represents the mean ± SE (n = 10). The asterisks over the bars represent statistical significance at * *p* = 0.05, ** *p* = 0.01, *** *p* = 0.001 for the Dunnett test.

A subsequent recovery of these parameters to those in intact control levels was observed in all plants at 110 dad, which was particularly evident in 100% defoliated *Tarasca* plants which developed significantly thicker stems at 110 dad (Figure 1 D). Dry panicle weights were unaffected by defoliation, irrespective of its severity (Figure 2 A), whereas panicle indexes and seed yields showed a tendency to increase in defoliated plants, with the former being significantly higher in 100% defoliated plants (Figure 2 B and C). Grain traits, such as seed weight, a parameter that tests seed quality, and germination efficiency were not affected by the defoliation treatments (Figure 3 A and data not shown). On the other hand, fully defoliated plants produced seeds having equal or higher starch contents (Figure 3 B).

### Differential responses to defoliation in grain amaranth in the greenhouse and field

**Figure 4:**
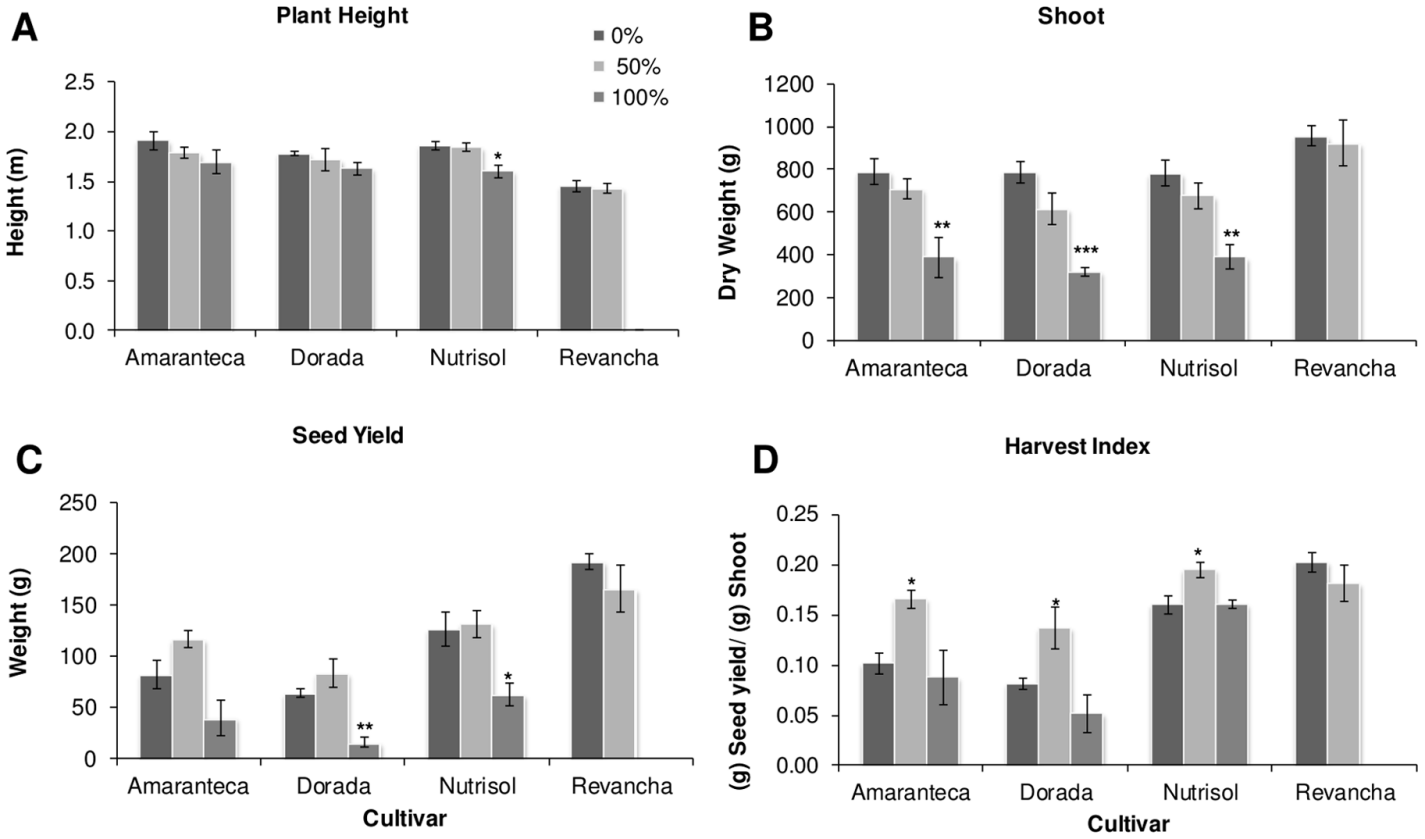
Effect of defoliation on grain amaranth growth and yield in field. Plants from different cultivars (*Amaranteca*, *Dorada*, *Nutrisol* and *Revancha*) were grown equally in the field and at panicle emergence were subjected to 3 defoliation treatments: control (0%), 50%, and 100% defoliation. Phenological parameters were measured at physiological maturity: A plant height; B shoot dry weight; C seed yield and D harvest index. Each bar represents the mean ± SE (n = 5). The asterisks over the bars represent statistical significance at * *p* = 0.05, ** *p* = 0.01, *** *p* = 0.001 for the Dunnett test.

**Figure 5:**
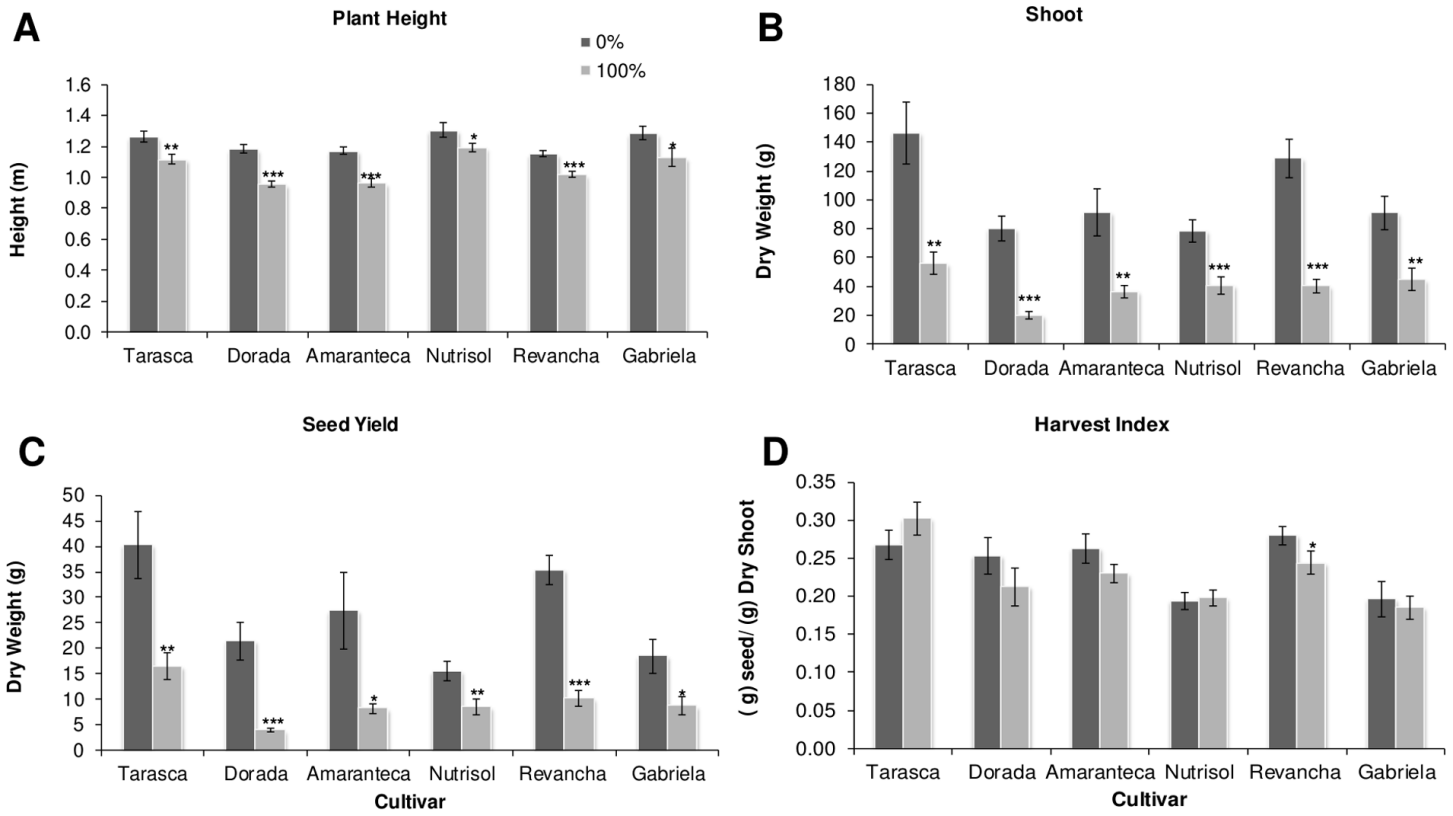
Effect of defoliation on grain amaranth growth and yield in green house. Plants from different cultivars (*Tarasca, Dorada Amaranteca,n utrisol, Revancha* and *Gabriela* ) were grown equally in the green house and at panicle emergence were subjected to 2 defoliation treatments: control (0%) and 100% defoliation. Phenological parameters were measured at physiological maturity: A plant height; B shoot dry weight; C seed yield and D harvest index. Each bar represents the mean ± SE (n = 10). The asterisks over the bars represent statistical significance at * *p* = 0.05, ** *p* = 0.01, *** *p* = 0.001 for the Dunnett test.

Additional field and greenhouse experiments were performed in the year 2011. Four of the most commercially important grain amaranth varieties cultivated in México, namely “*Amaranteca”*, “*Dorada*”, “*Nutrisol*” and “*Revancha*” were tested in the field. These materials, in addition to “*Tarasca*” and “*Gabriela*”, constitute the core of grain amaranth varieties recommended for cultivation in the diverse climates of Mexico [45]. The latter two varieties were also included in a simultaneous greenhouse experiment (see below). In the field experiments, plants were subjected to 50 and 100% defoliation (Figure 4), whereas in the parallel greenhouse experiment, only 100% defoliation treatments were applied (Figure 5). Interestingly, the effects of 100% defoliation on plant growth and reproductive fitness were more severe when the experiments were performed in the greenhouse since all parameters analyzed, excluding harvest index, were negatively affected in all six varieties examined (compare Figure 4 and Figure 5) One important difference with the previous three greenhouse experiments performed with “*Tarasca*” plants only (see Figure 1 to Figure 3), was that defoliation was done by cutting of the leaf apex and not by perforation. These results strongly suggest that the procedure employed to remove leaf area can significantly influence the effect of defoliation on the plant. Conversely, 100% defoliation in the field had not such a drastic effect, even though leaf removal was performed identically as the greenhouse experiments. Such outcome was somehow expected considering that greenhouse and field experiments have often been reported to yield contrasting results.

Nevertheless, total dry shoot mass and seed yield were negatively affected in several varieties examined in the field trials at 100 defoliation (Figure 4 B and C) whereas plant height was unaffected, except for cultivar Nutrisol, which was slightly but significantly diminished (Figure 4 A). Curiously, at 50% defoliation, three genotypes tested had significantly higher HIs than controls (Figure 4D). No comparisons at the 100% defoliation level could be made with the “*Revancha*” cultivar, which became highly susceptible to root rots by unidentified soil pathogens as the result of total leaf loss and perished.

**Figure 6:**
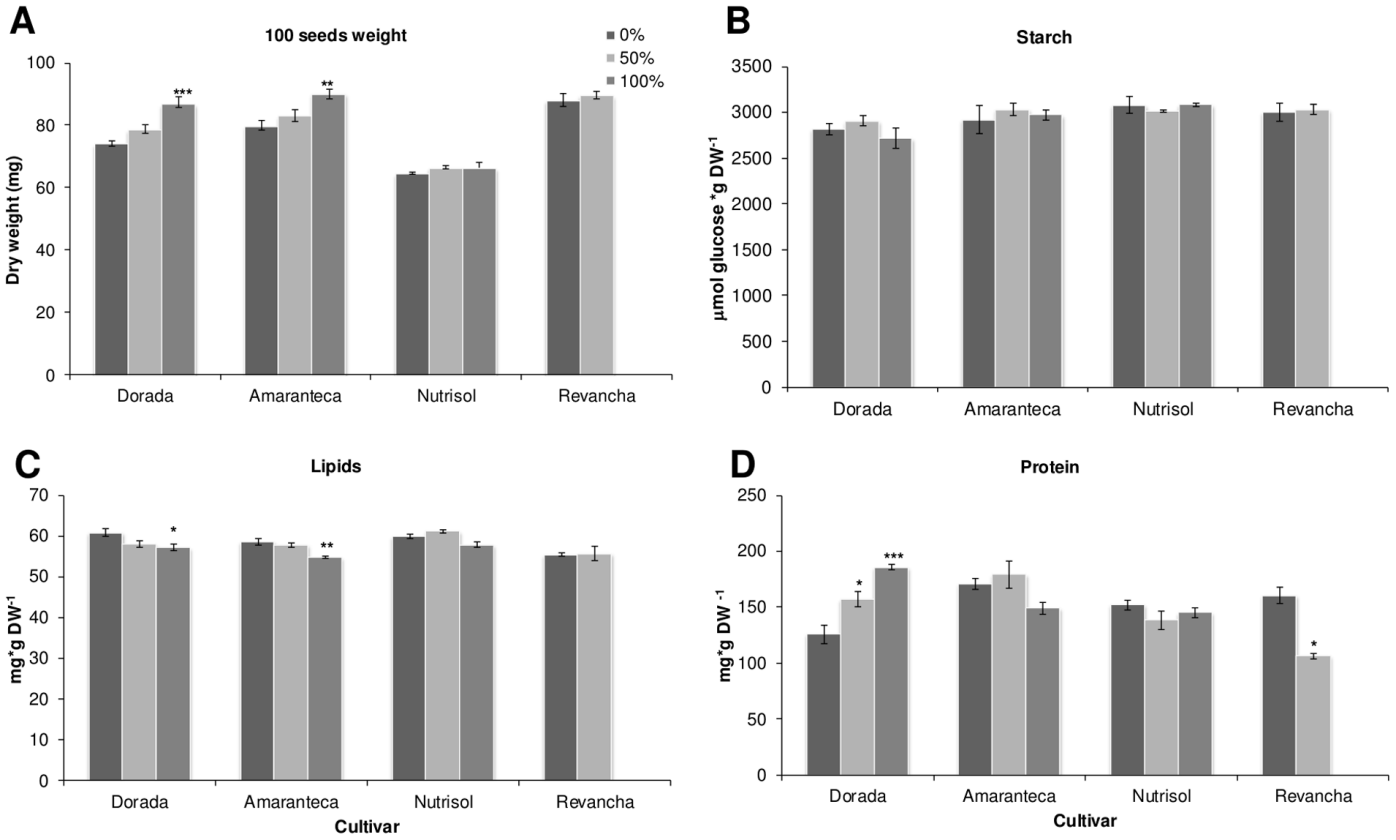
Seed composition in defoliated amaranth in the field. At mature amaranth seeds were collected and analyzed for A 100 seeds weight, B seed starch, C seed lipids and D seed protein. Each bar represents the mean ± SE (n = 5). The asterisks over the bars represent statistical significance at * *p* = 0.05, ** *p* = 0.01, *** *p* = 0.001 for the Dunnett test.

**Figure 7:**
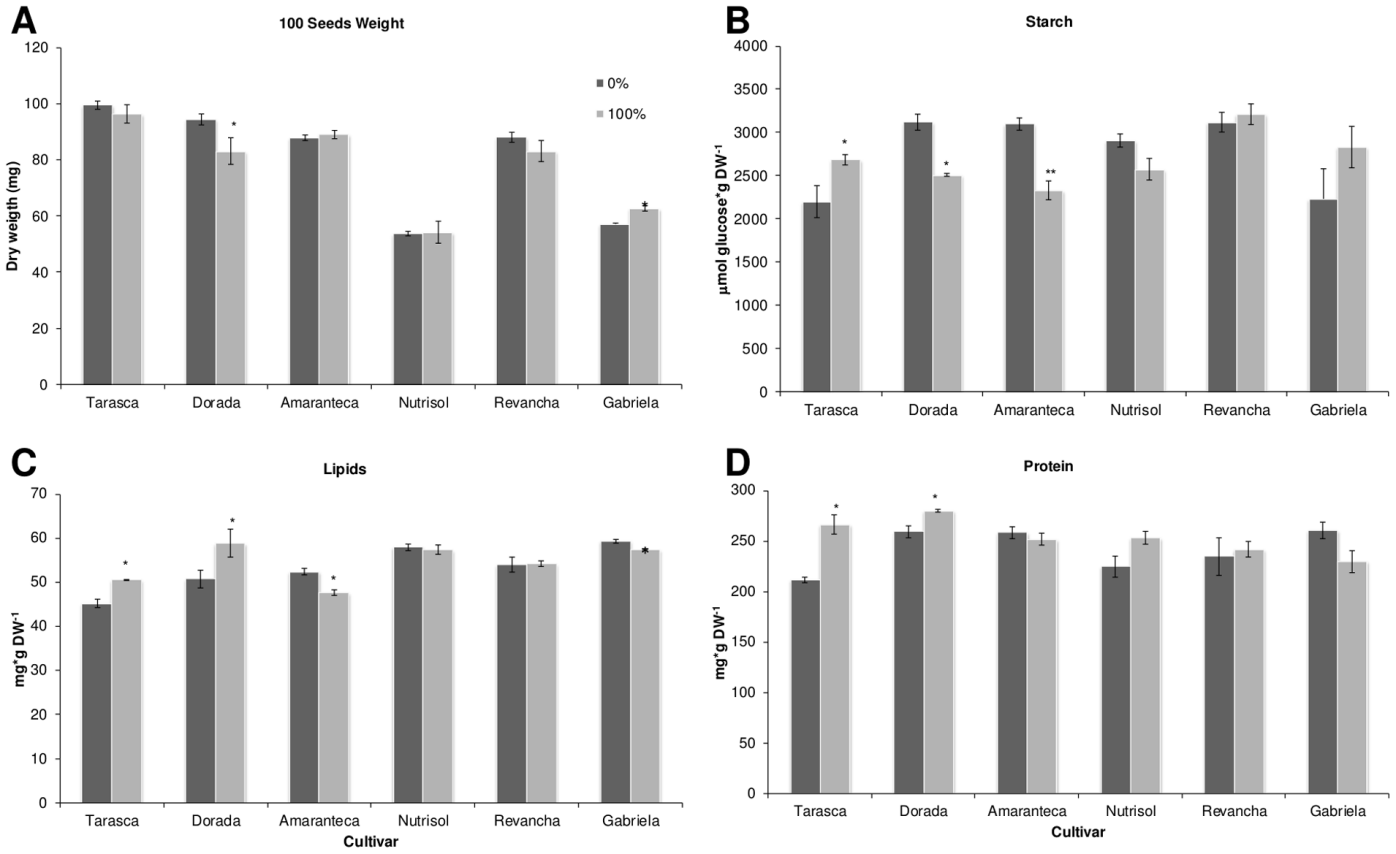
Seed composition in defoliated amaranth in green house. At mature amaranth seeds were collected and analyzed for A 100 seeds weight, B seed starch, C seed lipids and D seed protein. Each bar represents the mean ± SE (n = 10). The asterisks over the bars represent statistical significance at * *p* = 0.05, ** *p* = 0.01, *** *p* = 0.001 for t Test.

Interestingly, a significant effect on seed composition was produced by the experimental conditions in those varieties grown both in the field and in the greenhouse being higher in those produced in the greenhouse (starch, F = 4.1; 1 df; *P* = 0.05; lipid, F = 11.1; 1 df; *P* = 0.0019, and protein, F = 264; 1 df; *P*<0.0001; Compare Figure 6 and Figure 7). Several differences with the first defoliation by perforation experiments performed with the *Tarasca* cultivar under greenhouse conditions were also observed in all seed parameters examined (Compare Figure 3 with Figure 6 and Figure 7).

### Carbohydrate changes in stems and roots after defoliation

**Figure 8:**
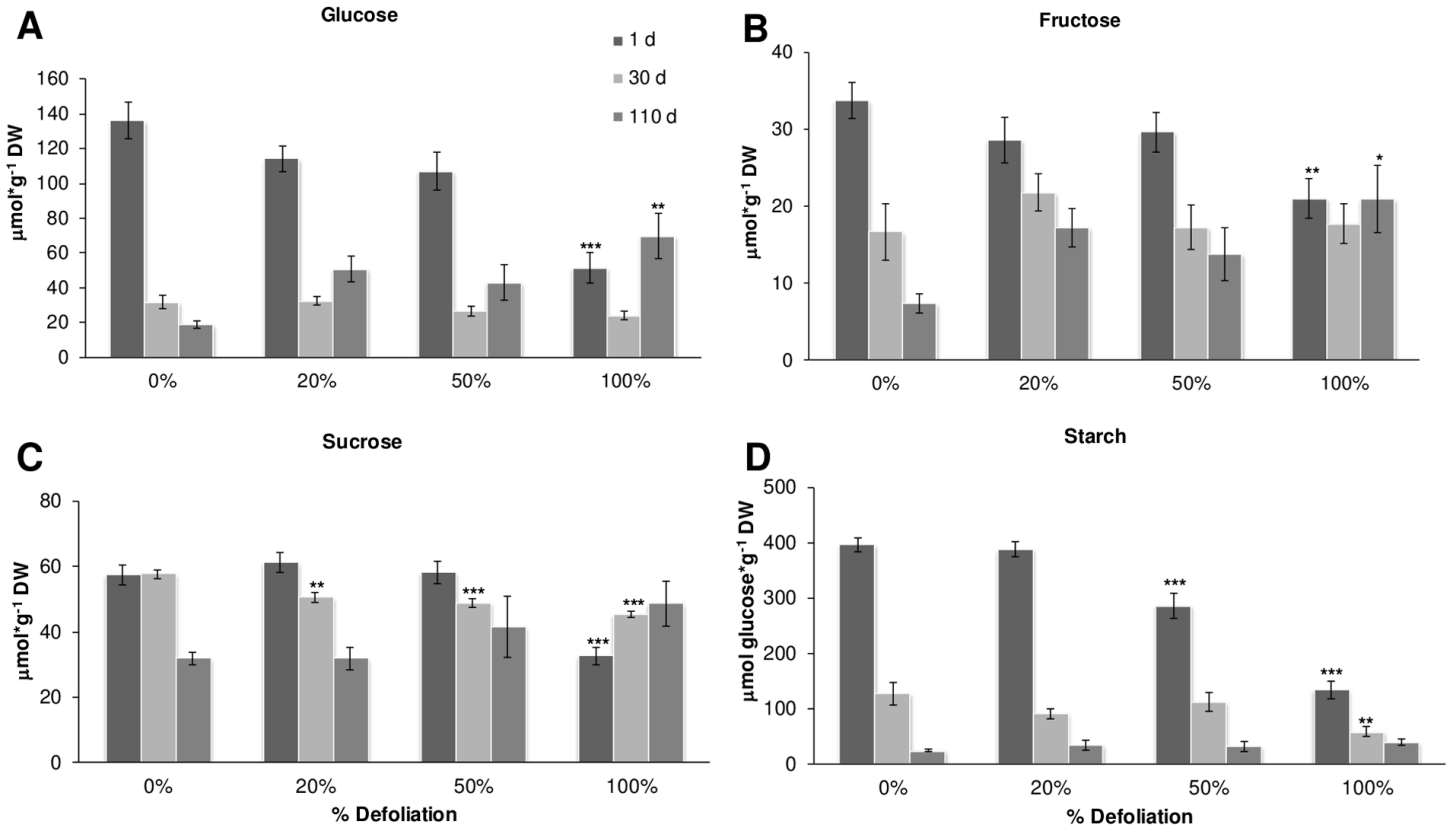
Carbohydrate levels in the stem of defoliated and undamaged amaranth plants. Plants were harvested at 1, 30 and 110 days after treatment. A glucose, B fructose, C sucrose and D starch. Each bar represents the mean ± SE (n = 10). The asterisks over the bars represent statistical significance at * *p* = 0.05, ** *p* = 0.01, *** *p* = 0.001 for Dunnett Test.

**Figure 9:**
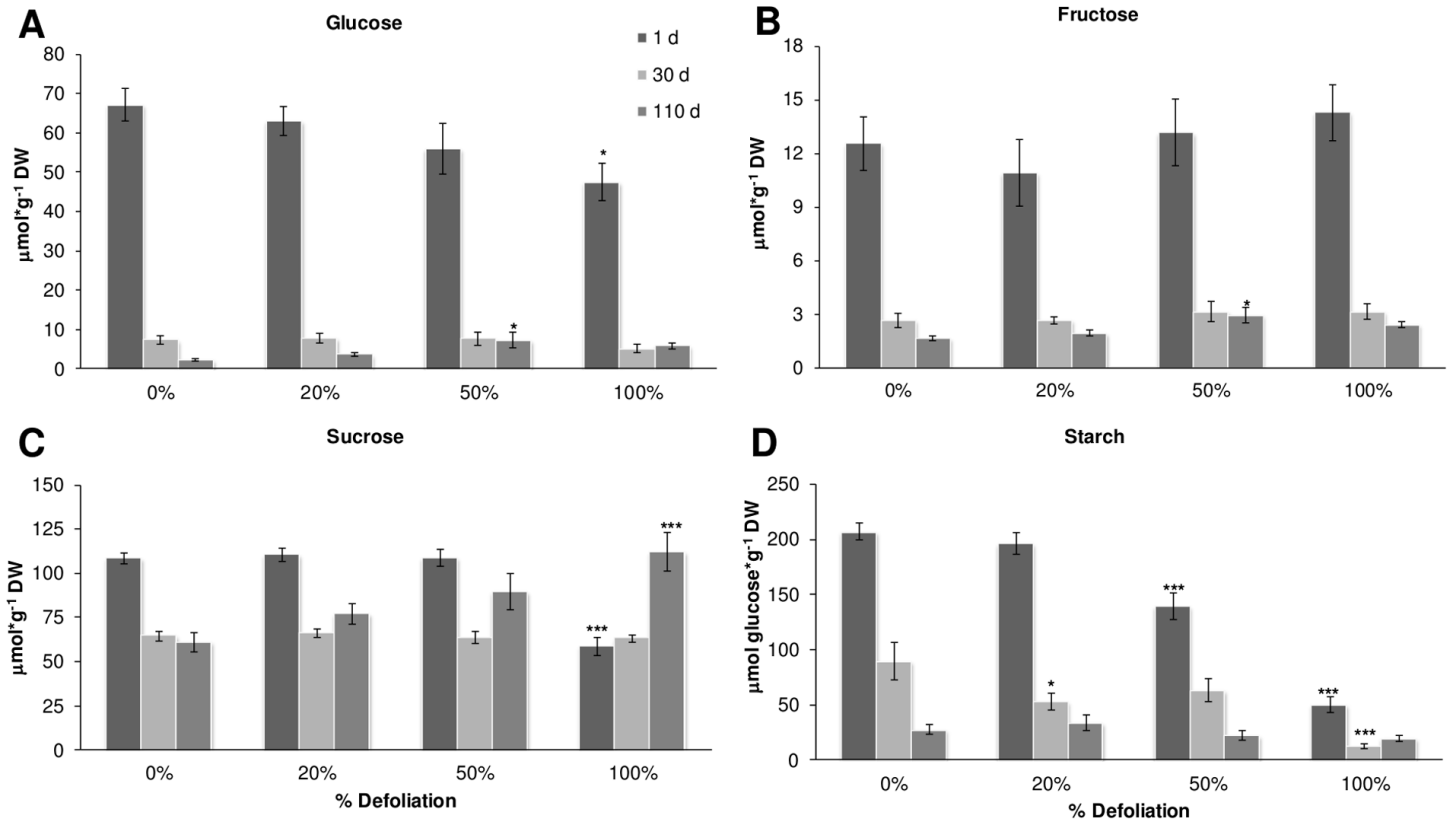
Carbohydrate levels in the roots of defoliated and undamaged amaranth plants. Plants were harvested at 1, 30 and 110 days after treatment. A glucose, B fructose, C sucrose and D starch. Each bar represents the mean ± SE (n = 10). The asterisks over the bars represent statistical significance at * *p* = 0.05, ** *p* = 0.01, *** *p* = 0.001 for Dunnett Test.

Although all nonstructural carbohydrates (NSC) were analyzed, the measurement of starch and SUC was of particular importance considering that SUC-starch metabolism is a major determinant of sink strength, C partitioning and HI [46]. NSC were measured in stems and roots of control and defoliated amaranth plants at 1, 30 and 110 dad. NSC levels in leaves were not analyzed considering that leaf sampling was impossible in the case of 100% defoliation treatments, at least at 1 dad. In the greenhouse experiments with “*Tarasca*” plants, defoliation severity affected the NSC analyzed in different ways. In stems, SUC level (∼60 μmol/g DW in controls at 1 dad), changes were dependent on the defoliation severity since reductions were observed at 30 dad in 20 and 50% defoliated plants, while 100% defoliation accelerated SUC decline to 1 dad (Figure 8 C). In roots, (∼110 μmol/g DW in controls at 1 dad), a similarly rapid reduction of SUC was observed at 1 dad in 100% defoliated plants (Figure 9 C). In both stems and roots, starch levels, which constitute the most abundant NSC in amaranth (∼200 and ∼400 μmol/g DW in controls at 1 dad, respectively), were rapidly reduced at 1 dad in 50 and 100% defoliated plants, although the latter treatment extended this decline to 30 dad (Figure 8 and Figure 9 D). Glucose (GLC), the next most abundant NSC in stems and roots (∼70 and ∼140 μmol/g DW in controls at 1 dad, respectively) was rapidly reduced in both tissues as plants developed. However, the reductions became significant only in 100% defoliated plants at 1 dad, and were stronger in stems (Figure 8 and Figure 9 A). At 110 dad, a significant accumulation of GLC and FRC (∼30 and ∼10 μmol/g DW in controls at 1 dad, respectively), in stems of 100% defoliated plants (Figure 8 A and B), and SUC, in roots of 100% defoliated plants (Figure 9 C) was observed. The above results indicate that starch and SUC reserves in the stems and roots of amaranth can function as a C reserve to buffer the immediate effects of severe defoliation.

### Amaranth's response to shading

**Figure 10:**
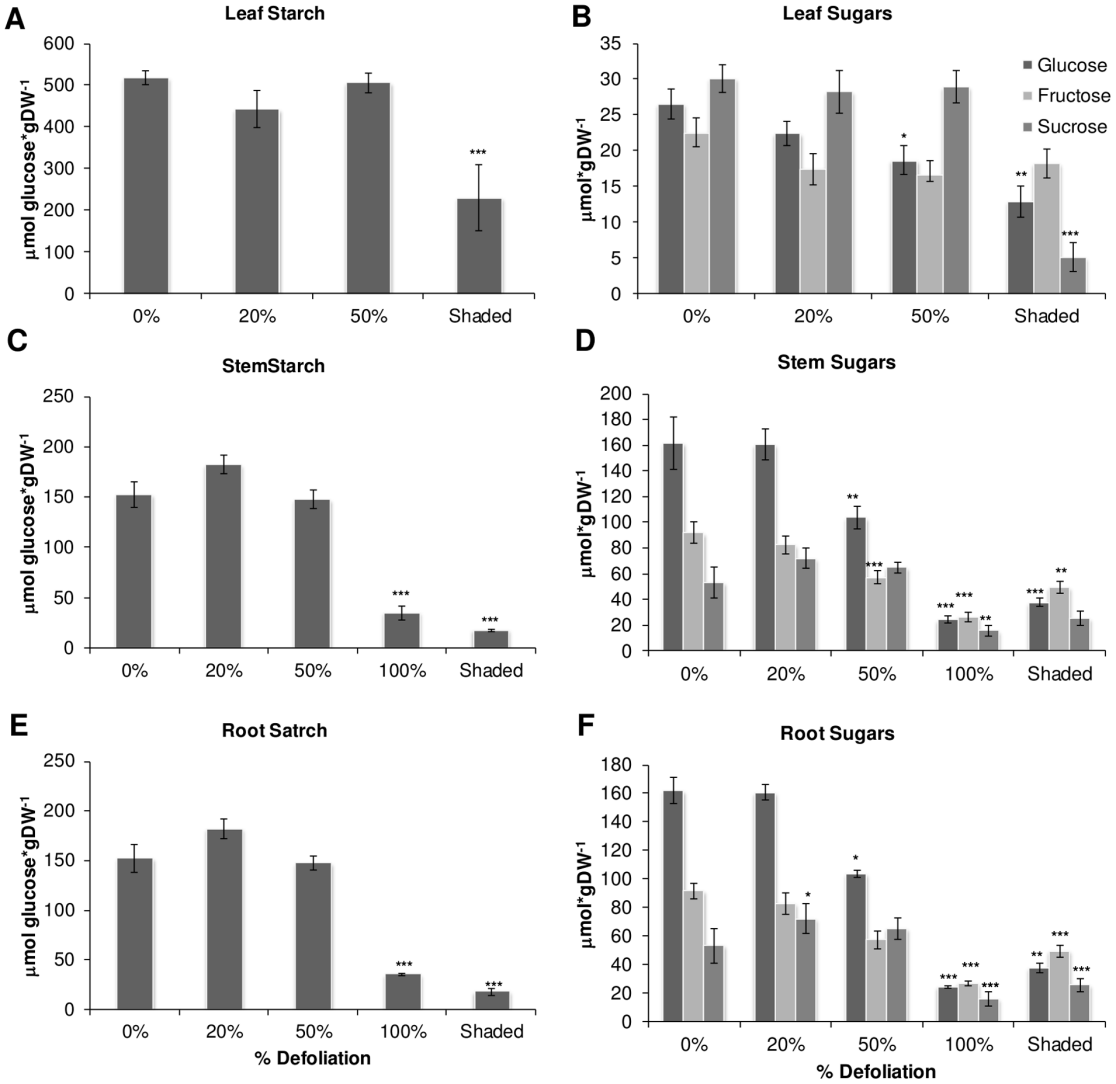
Carbohydrate levels in defoliated and shaded amaranth plants at 1 day after treatment. Starch levels in A leaf; C stem and E root. Sugars levels in B leaf; D stem and F root. Each bar represents the mean ± SE (n = 10). The asterisks over the bars represent statistical significance at * *p* = 0.05, ** *p* = 0.01, *** *p* = 0.001 for Dunnett Test.

The above results lent support to the proposed mechanism by means of which active mobilization of C stores in stem and roots of heavily defoliated amaranth plants act as a buffering mechanism to compensate for leaf-loss imposed by mechanical damage. Leaf shading is another commonly used method to manipulate C-allocation responses [47] that has the advantage of limiting photosynthesis without damaging the leaves. It was also employed in this study to determine the short-term effect of resource limitations due to reduced photosynthesis on the mobilization of stored C resources in stems and roots of grain amaranth. Not surprisingly, the results obtained closely resembled those obtained at 1 dad in 50% and 100% defoliated “*Tarasca*” plants, denuded by leaf removal. Thus, all NSC levels, including those in leaves (except FRC) were significantly reduced (Figure 10). These results reinforced the proposal that when C acquisition by photosynthesis is affected or abolished in defoliation tolerant plants, such as grain amaranth, the energy requirements for regrowth and/or normal functioning will be supplied from stored C reserves.

### Changes in sucrolytic and amylolytic activity in shaded or defoliated plants

**Figure 11:**
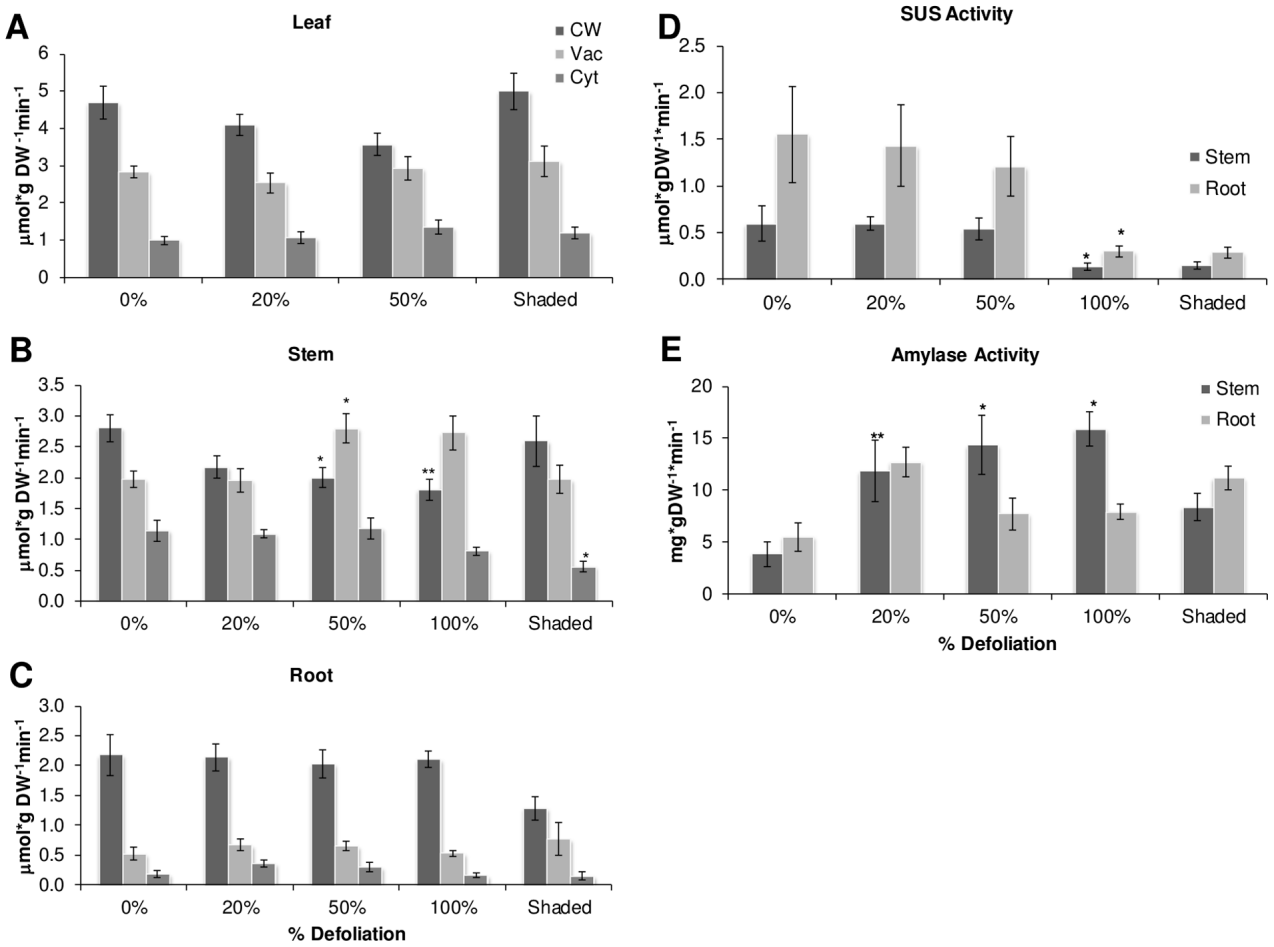
Sucrolytic and amylolytic activity in defoliated and shaded amaranth plants 1 day after treatment. Invertase activity in A leaf, B stem, C Root. D SUS activity in root and stem. E total amylase activity in stem and root. Each bar represents the mean ± SE (n = 10). The asterisks over the bars represent statistical significance at * *p* = 0.05, ** *p* = 0.01, *** *p* = 0.001 for Dunnett Test.

Sucrose synthases (SUS) (EC 2.4.1.13) and invertases (EC 3.2.1.26, β-fructosidase, β-fructofuranosidase) are enzymes that play numerous roles in plants, including sugar import, carbon partitioning and establishment of sink strength. The effect of defoliation or shading of the activity of SUS in stems and roots was greatly affected by 100% defoliation at 1 dad and by shading (Figure 11 D). All other sucrolytic activities analyzed were affected in stems of defoliated plants only. Thus CWI activity was significantly reduced by defoliation, irrespective of the intensity of foliar loss, at 1 dad (Figure 11 A). On the other hand, cytosolic invertase activity was decreased in 100% defoliated plants and in shaded plants at 1 dad (Figure 11 B), whereas no changes in vacuolar invertase activity were detected, although it tended to increase in shaded plants (Figure 11 C). No differences with controls were observed in sucrolytic activity measured at 30 dad (data not shown). On the other hand, the reduction of stem and root starch reserves observed predominantly at 1 dad, correlated with an increase of total amylolytic activity, as shown in Figure 11 E.

## Discussion

Removal of specific green tissues or inhibition of photosynthesis (e.g. defoliation or shading) causes altered sink-source relationships and is therefore a useful model to study the physiological adaptations of plants in response to these particular stress conditions. Defoliation limits the production of exportable sugars (mainly SUC) which are required as a fuel for meristematic activity and for the growth of sink organs such as roots, new leaves, flowers, fruits and seeds.

Amaranth is considered to be a plant species that can tolerate herbivore pressure frequently associated with extensive defoliation, although solid experimental data to support this claim is still scant. Until recently, the underlying mechanism(s) for this property in amaranth remained unknown, although previous studies performed with *A*. *hybridus* suggested that pre-flowering allocation of resources to the taproot, which in other species greatly contributes to the storage of C and N, was proposed to be the primary mechanism by which *A. hybridus* was able tolerate folivory [48], [49]. However, the recent developments represented by this work, complement and expand a parallel study showing that tolerance to partial (∼30%) defoliation in *A. cruentus* by either insect herbivory or mechanical damage was likely associated to a rapid utilization of C reserves. In the latter study, these were proposed to be predominantly in the form of foliar starch, although mobilization of C reserves in stems and roots was also observed [20], since leaf area was not so extensively eliminated by extensive defoliation treatments as in this study. Here, we propose that it was mostly starch reserves in stems and roots that were mobilized to sustain recovery and productivity after the drastic levels of defoliation applied (Figure 8 and Figure 9). This concept implies that in grain amaranth, tolerance to defoliation is based on an adjustment of C allocation to different organs according to priorities of vegetative growth and reproductive development.

However, in both studies, a long-term replenishment of C reserves after defoliation was also observed at 110 dad, with significant increases of some sugars, such as SUC in roots and GLC and FRC in stems (Figure 8 and Figure 9). This is a characteristic that is also considered to enhance tolerance to leaf loss in highly grazing-tolerant grasses such as *Lolium perenne* [21]. Thus, the observed utilization of stored C reserves in response to defoliation in grain amaranth was in agreement with findings in many defoliation and grazing-tolerant species in which the reduced photosynthetic activity and energy supply occurring as a consequence of a drastic to total reduction of leaf area, generally activated a quick mobilization of C reserves, predominantly as starch, to sustain regrowth and ensure survival [21], [50], [51], [52]. It was also in agreement with data from other studies that have shown that stored resources play an important role in reducing the net seasonal physiological costs of reproduction in tolerant plants when leaf photosynthetic activity is manipulated, either by shading or defoliation (see Figure 10, for example) [47], [53], [54]. Therefore, our results strongly suggest that grain amaranth appears to have sufficient resource supplies, i.e. starch, to cover the C/energy demand imposed by reproduction and the needs of other sinks, and may explain the observed general insensitivity to defoliation that reproductive fitness, measured as seed productivity, has in grain amaranth.

Results of sucrolytic acivities (Figure 11) support the findings reported by the above previous study in which it was shown that grain amaranth can alter the activity/expression of key carbohydrate (CHO)-related enzymatic activities and genes after partial leaf-loss in order to ensure the rapid and sometimes sustained hydrolysis, mobilization and utilization of carbohydrate reserves [20]. In the study herewith presented, SUS activity in stems and roots was found to be drastically and rapidly reduced, one day after complete defoliation or shading. This fall probably indicated that roots and stems underwent a shift from sink to source tissues. This is consistent with the fact that SUS, which catalyze the reversible cleavage of SUC to UDP-glucose and FRC are known to be predominately involved in sugar import and establishment of sink strength [55], [56]. The early decrease in cell wall invertase activity, although not as drastic as SUS, was also in agreement with these tissues undergoing a sink to source transition as a result of severe defoliation [57]. On the other hand, increased amylase activity at 1 dad was consistent with the rapid changes occurring in starch reserves, particularly in stems. It was also in agreement with a previous study in which amylase activity and ß-amylase expression were rapidly induced in response to partial defoliation in *A. cruentus* [20].

In fact, the results obtained indicate that the strategy to tolerate defoliation used by amaranth is effective enough to ensure that the reduction in seed production as a result of total leaf loss is minimal or avoided, except for certain genotypes and experimental conditions. The physiological mechanism involves starch degradation in stem and roots and SUC relocation for providing carbon for regrowth. It is proposed that once new shoot branches and leaves are formed using those internal reserves, increased photosynthetic area allows the formation of reproductive organs, which then produce seeds.

However, it must be noted that the way defoliation was performed in grain amaranth had a very significant effect on its ability to tolerate leaf loss. This was clearly observed in the greenhouse experiments in which defoliation was done by perforation (see Figures 1 to 3). This form of producing leaf tissue loss was highly tolerated, compared to another in which defoliation was done by cutting, starting from the apex of the leaf (see Figure 4 and Figure 5), which proved to have a more deleterious effect on the plant in terms of growth and reproductive parameters. This was in agreement with previous observations indicating that the way leaf tissue is removed from the plant can have a profound effect on its physiology and even defense-responses. For instance, partial defoliation by mechanical damage or insect herbivory profoundly affected CHO- and defense-related gene expression in grain amaranth although the tolerance to defoliation was similar [20]. It has also been shown that the degree of photosynthetic impairment caused by insect herbivory in Arabidopsis is higher when damage is caused by first instar larvae, which typically make small holes and avoid veins, than that produced by older larvae [58]. Also, chewing damage in soybean caused by skeletonizing Mexican bean beetles (*Epilachna varivestis*) can cause substantial losses of photosynthesis in the remaining leaf tissue in contrast to a similar extent of damage caused by larvae of other chewing insects. In all these cases the difference was associated to an exacerbated localized water stress, leading to tissue desiccation and photosynthesis repression [15]. Moreover, a recent study showed that leaf-edge removal, compared to leaf-apex removal and perforation was the only simulated-herbivory method that was able to reduce the total plant and root biomass of *Ipomea cairica*, an undesired invasive vine [59]. The differential effect was proposed to be influenced to a certain degree by the particular leaf anatomy of this plant species, which suffered maximal damage when the leaf edges were removed. This suggests that certain type of insects, e.g. large caterpillars, whose feeding habit of removing parts of or entire leaves can be simulated by clipping [60], could have the greatest negative effect on productivity in grain amaranth crops, compared to younger instars or insects belonging to other feeding guilds. This potentially important agronomic aspect remains to be determined.

Another important finding of this study was that tolerance to defoliation in grain amaranth appears to be genetically determined, as it varied amongst species and amongst varieties within species. This was more clearly shown in the field experiments. First, the two *A. cruentus* varieties tested whose productivity tended to be more negatively affected by defoliation than “*Nutrisol*”, the only *A. hypochondriacus* variety that survived the 100% defoliation treatment (Figure 4). Second, “*Revancha*” the second *A. hypochondriacus* variety employed in these trials was unable to survive the 100% defoliation treatment, thereby suggesting that total loss of foliar tissue greatly increases its susceptibility to soil borne pathogens leading to root rots. Several explanations for the negative effect that defoliation had on this particular variety may be given. Thus, the defoliation-induced susceptibility could have had a genetic origin since “*Nutrisol*” and “*Revancha*” belong to two different races (“Azteca” and “Mercado”, respectively) having distinctive characteristics [45]. This could have had influenced the way CHO storage in stems and roots occurs in these varieties, or indicated a different allocation of C stores to development and defense. For instance, in neo-tropical forests, differences in CHO storage are believed to be a factor influencing differences in seedling survival between species following defoliation [61].

In other studies, defoliation-induced susceptibility to disease or increased root herbivory has been attributed to differences in the induced synthesis of C-rich chemical defenses in roots, such as phenols and terpenoids [22], [62], [63]. Another explanation could be that decreased C content of stems and roots lead to disturbances in the nutrient uptake by roots [64], or that an excessive cost of new leaf production following defoliation caused a reduction in root mass and increased root mortality [64], [65]. In this respect, it was found from field observations that sugar beets (which are closely related to grain amaranth) defoliated by water stress showed an increased susceptibility to root rotting after water supply restoration. A study performed to analyze this phenomenon found that, similarly to what was found with the “Revancha” cultivar, the number of rotted roots increased concomitantly with the defoliation level, reaching significant levels only at severe levels of defoliation. Another similarity with our study was that differences in susceptibility to root rots, which were attributed to several fungi varied among the three commercial cultivars examined [66]. However, the reason(s) why the “*Revancha*” cultivar and perhaps other cultivars belonging to the same “Mercado” race are susceptible to root rots when severely defoliated remain to be determined. Also, it is not known whether the “*Revancha*” cultivar is unsuitable for cultivation where herbivory pressure and soil moisture are very high.

It was also interesting to observe that seeds produced by plants subjected to different forms of defoliation or defoliated under different environments (e.g. field vs. greenhouse) showed different compositions and even different size (see Figure 6 and Figure 7 and data not shown). Again, defoliation by cutting had a more drastic effect than perforation, especially if the damage was produced in the greenhouse, and the modifications were more frequently observed in *A. cruentus* plants. Differences between field and greenhouse experiments are not rare and have been observed when diverse anatomical and physiological plant parameters have been tested such as leaf characteristics in cotton [67], photosynthesis efficiency and/or activity in *Populus* and birch [68], [69], environmental effects on fungal resistance in genetically modified wheat [70] phenolic accumulation and polyphenol oxidase activity in tobacco [71] and pod dehiscence in soybean [72].

Also, changes in seed composition due to defoliation appear to be a rather common phenomenon since, similarly to grain amaranth, loss of leaf tissue has been observed to lead to changes in composition, size or yield in many plant species [73], [74].

It is concluded that grain amaranth is highly tolerant to defoliation. This trait appears to be related to the efficient use of C reserves, mostly as starch, stored in stems and roots, whose mobilization to different organs is adjusted according to priorities of vegetative growth and reproductive development. Complete defoliation and shading had similar effect on the C reserves of these tissues, whose sink to source transition was accompanied by a sharp decrease in SUS activity in both stem and roots and lower levels of cell wall invertases in stems. Tolerance to defoliation in grain amaranth was highly dependent on the amount of damage done, the way defoliation was performed, and on the environment in which defoliation was produced, with cutting having a more deleterious effect than perforation, particularly in greenhouse conditions. A genetic effect was also observed, with *A. cruentus* plants generally undergoing more changes as a result of leaf loss than *A. hypochondriacus*, although one *A. hypochondriacus* cultivar tended to suffer lethal effects when completely defoliated.
